# Workplace Vaccination and Other Factors Impacting Influenza Vaccination Decision among Employees in Israel

**DOI:** 10.3390/ijerph7030853

**Published:** 2010-03-08

**Authors:** Shosh Shahrabani, Uri Benzion

**Affiliations:** 1 The Economics and Management Department, The Max Stern Academic College of Emek Yezreel, Emek Yezreel 19300, Israel; 2 Department of Economics, Ben-Gurion University, Beer-Sheva 84105, Israel; 3 Western Galilee College, P.O.B. 2125, Akko 24121, Israel; E-Mail: uriusa2@gmail.com

**Keywords:** influenza vaccination, health belief model, employees

## Abstract

The study examined the factors affecting the decision to be vaccinated against influenza among employees in Israel. The research, conducted in 2007/2008, included 616 employees aged 18−65 at various workplaces in Israel, among them companies that offered their employees influenza vaccination. The research questionnaire included socio-demographic characteristics, and the Health Belief Model principles. The results show that the significant factors affecting vaccination compliance include a vaccination program at workplaces, vaccinations in the past, higher levels of vaccine’s perceived benefits, and lower levels of barriers to getting the vaccine. We conclude that vaccine compliance is larger at companies with workplace vaccination programs providing easier accessibility to vaccination.

## 1. Introduction

Influenza is a prevalent and highly contagious disease that each year results in increased morbidity and mortality on a global scale. Because of the widespread nature of this disease, annual influenza epidemics cause substantial workplace absenteeism, and the associated cost of lost productivity is a significant component of the considerable financial burden this disease places on employers and on society [1–3]. Workplace vaccination programs against influenza have been found to be cost-effective from the points of view of society and of employers [4–6]. Nevertheless, to the best of our knowledge the effect of workplace vaccination programs on vaccination compliance among employees has not yet been examined. The current study fills this void.

This study empirically examined the factors affecting: (a) the decision to be vaccinated against influenza in 2006/2007, and (b) the intention to be vaccinated in the coming 12 months among employees in Israel. The study focused on employees at workplaces that offered the vaccine to their employees and on those that did not offer their employees the vaccine. More specifically, we tested the following:

- The impact of offering vaccination at workplaces on the decision to be vaccinated.
- The impact of several other factors on the decision to be vaccinated. These factors include the Health Belief Model (HBM) [7] categories, such as perceived susceptibility to influenza, perceived severity of influenza, perceived benefits of the vaccine, and barriers to getting vaccinated. In addition, we examine socio-demographic characteristics, and personal factors such as perceived heath status, perceived risk of illness, and perceived vaccine cost.
- Main reasons for accepting or rejecting flu shots by employees in 2006/2007.

The paper is organized as follows: Section 2 presents the literature review, Section 3 describes the model, and Section 4 describes the methods. Section 5 presents the major results, and Section 6 summarizes the conclusions.

## 2. Literature Review

Several recent studies have evaluated the economic burden of influenza on society, taking into account workplace absenteeism and the associated costs of lost productivity [1–3,8]. For example, in 2008 Keech and Beardsworth reviewed a number of studies in an attempt to quantify the impact of influenza upon otherwise healthy adults in terms of lost work days associated with an influenza episode. These studies, which involved study sites in North America, Western Europe, Asia and Australia, generally showed that the mean number of work days lost ranged between 1.5 and 5.9 days per influenza episode. The review highlights the significant economic impact of influenza, *i.e.,* the loss of productivity caused by absenteeism as well as by employees functioning at reduced capacity even after they returned to work.

Influenza vaccination has been shown to be cost effective in reducing morbidity, work absenteeism, and use of healthcare resources among the healthy working adult population [8–10]. Vaccine prevents influenza in approximately 70%–90% of healthy adults under the age of 65 [9,11]. Several studies evaluating the health and economic benefits of a workplace vaccination program against influenza show that workplace vaccination of healthy adults against influenza has a clear impact on rates of influenza-like illness (ILI), absenteeism, and reduced company productivity. These health benefits translate into financial benefits for the employer, with cost savings significantly outweighing the costs of the vaccination program [4–6,12].

Based on several models published in the literature, Olsen *et al.* (2005) [5] calculated that estimated savings per healthy working adult employee could be anticipated to range from $15 to $50 US. Variations in this net savings are based primarily on assumptions of employee productivity estimates. In addition, efficient influenza immunization programs at large worksites are feasible and worthy of employer consideration for economic reasons as well as for reasons of employee satisfaction.

Several studies have examined factors affecting the decision to get the flu vaccine using the Health Belief Model (HBM) [7] as a conceptual framework to examine preventive behavior (e.g., vaccination). The HBM explains and predicts preventive health behavior in terms of belief patterns focusing on the relationship between health behaviors and utilization of health services. According to the HBM, getting vaccinated against influenza depends on the following predictors: perceived susceptibility to influenza, beliefs about severity of influenza, perceived benefits of the vaccine in preventing influenza, and perceived barriers to getting vaccinated [13–15]. Indeed, cited reasons for not getting influenza vaccination were similar across studies with respect to perceived barriers, *i.e.*, concern about side effects or vaccine safety, lack of vaccine effectiveness in preventing illness, and lack of awareness [13,15–18]. Our study is partially based on the HBM framework as implemented for employees in Israel.

Socio-demographic background, economic status, and health status are also known to have an impact on an individual’s decision to get vaccinated [19,20]. In an empirical study conducted in the USA, Wu (2003) found that people with more education, higher incomes, and better insurance coverage are more likely to get flu shots, as well as various other types of preventive medical treatments. In addition, several studies have shown that individuals’ risk perceptions predict their subsequent vaccination against influenza, meaning that a higher perceived likelihood of becoming ill is associated with greater tendencies to get vaccinated [21–23]. It was also found that past experience with influenza vaccination is a predictor for willingness to get vaccinated [24], since those who were vaccinated previously may continue to do so annually as a matter of routine.

The current study examined the factors affecting the decision to get vaccinated against influenza among employees in Israel. In particular, we examined the impact of offering vaccination at workplaces on the decision to be vaccinated.

## 3. The Model

Using regression equations for the analytical model, we examined the factors affecting the vaccination status against influenza in 2006/2007 and the intention to be vaccinated in the coming 12 months. The analytical model examines the effect of each one of the explanatory variables on the dependent variables, controlling for all other variables including socio-demographic characteristics.

The following equations describe the analytical model:

(1) $$\begin{array}{l} {\text{Y}}_{1}=\alpha +\beta_{1}{\text{HBM}}_{1}+\beta_{2}{\text{HBM}}_{2}+\beta_{3}{\text{HBM}}_{3}+\beta_{4}{\text{HBM}}_{4}+\gamma_{1}\text{RISK}+\gamma_{2}\text{COST}\\ \;\;\;\;\;\;\;\;\;\;\;\;\;+\gamma_{3}\text{HEALTH}+\gamma_{4}\text{RECOM}+\delta_{1}\text{HMOTIV}+\delta_{2}\text{KNOW}+\delta_{3}\text{OFFER}+\lambda_{1}\text{Gender}\\ \;\;\;\;\;\;\;\;\;\;\;\;\;+\lambda_{1}\text{Age}+\text{u} \end{array}$$

(2) $$\begin{array}{l} {\text{Y}}_{2}=\alpha +\beta_{1}{\text{HBM}}_{1}+\beta_{2}{\text{HBM}}_{2}+\beta_{3}{\text{HBM}}_{3}+\beta_{4}{\text{HBM}}_{4}+\gamma_{1}\text{RISK}+\gamma_{2}\text{COST}\\ \;\;\;\;\;\;\;\;\;\;\;\;\;\;+\gamma_{3}\text{HEALTH}+\gamma_{4}\text{RECOM}+\delta_{1}\text{HMOTIV}+\delta_{2}\text{KNOW}+\delta_{3}\text{OFFER}+\lambda_{1}\text{Gender}\\ \;\;\;\;\;\;\;\;\;\;\;\;\;\;+\lambda_{1}\text{Age}+\text{u} \end{array}$$

In the first equation, the dependent variable, Y_1_, is the vaccination status in 2006/2007 (yes or no), and in the second equation, the dependent variable, Y_2_ is the intention to be vaccinated in the next 12 months (intend or not intend). The explanatory variables include:

*HBM categories:*

○ (a) HBM_1_—perceived *susceptibility to influenza:* Individuals at the low end of the susceptibility spectrum deny the possibility of contracting the illness, while those at the high end feel they are in real danger of contracting influenza.
○ (b) HBM_2_—perceived *severity of influenza*: This category describes the level of an individual’s beliefs concerning the potential difficulties caused by influenza, such as pain and discomfort.
○ (c) HBM_3_—perceived *benefits of the vaccine:* This category describes the level of an individual’s beliefs concerning what he or she stands to gain by getting the flu shot.
○ (d) HBM_4_—perceived *barriers to getting vaccinated*: This category describes the level of an individual’s beliefs concerning potential difficulties caused by the vaccine, such as inconvenience and unpleasantness.

We expected that intention to be vaccinated and vaccination status would be positively affected by higher levels of susceptibility, severity, and benefits (HBM1-HBM3), and negatively affected by higher levels of barriers (HBM4) [13]. Following previous studies [15,19,24] we added the following subjective and personal factors as explanatory variables:

*Subjective and personal factors*: RISK—perceived risk of infection if not vaccinated; COST— perceived cost of vaccination; HEALTH—perceived health status (bad, good); RECOM—whether or not the vaccine was recommended by physician, family or friends; HMOTIV—health motivation, referring to degree of motivation for other health behaviors; KNOW—knowledge about influenza and the vaccine; OFFER—whether or not the vaccine was offered at the workplace. We expected that higher levels of perceived infection risk, perceived cost of vaccination, health motivation, knowledge and vaccination recommendation would positively affect an individual’s intention to be vaccinated and his or her vaccination status (based on Shahrabani *et al.*, 2009 results with respect to nurses in Israel [15]). In addition, we expected the intention to be vaccinated and the vaccination status to be higher for employees whose workplaces offer the vaccine.
*Socio-demographic factors:* including gender and age groups.

## 4. Methods

### 4.1. Design

A cross-sectional design research methodology was adopted, covering the period from November 2007 to March 2008. The study population included 616 employees at various workplaces in Israel, ranging in age from 18 to 65 years old. We chose thirteen organizations from various industries in Israel. According to the main study question, we included two types of companies from each type of industry: companies that offered influenza vaccination to their employees in 2006/2007 and those that did not offer the vaccine to their employees. The organizations were: (a) five traditional industrial plants including the refinery complex, and the petrochemical complex, both offering the vaccine to their employees, the electricity company and the petrol and energy company, which did not offer the vaccine, (b) four service organizations including a higher education institution and an engineering services firm, both offering the vaccine, and an industrial supply services firm and another engineering services firm that did not offer the vaccine, (c) two large international high-tech organizations including optical products company, which offered the vaccine, and an information technologies company, which did not offer the vaccine. The study was approved by the Ethics Committee of the Max Stern Academic College of Emek Yezreel.

### 4.2. Measures

The research questionnaire was partially based on the questionnaire developed by Blue and Valley (2002) [13] and on its Hebrew version implemented for health care employees [15]. The final version of the questionnaire was finalized after analyzing data of a pilot questionnaire distributed at two workplaces.

The questionnaire consisted of the following parts: (1) items requesting socio-demographic information, including age, marital status, education, nationality, experience at work, and membership in a particular Health Maintenance Organization (henceforth, HMO); (2) whether the respondent had been vaccinated against influenza (yes or no) in the last year; (3) the intention to be vaccinated in the next 12 months, and the intention to be vaccinated if the flu shot is offered free of charge at the respondent’s place of work, on a 5-point scale ranging from 1 (“certainly I will get the vaccine in the next year”) to 5 (“I will definitely not get the vaccine in the next year”); (4) reasons for getting or not getting vaccinated, and place of vaccination for those who took the flu shot; (5) past flu vaccination history, and perceived health status (ranging from 1-“very good” to 4-“poor”); (6) perceived probability of contracting influenza without the vaccine and after getting the vaccine (5-point scale ranging from 1-“very high” to 5-“very low”); (7) items measuring the HBM variables, including the four categories of susceptibility, seriousness, benefits, and barriers, as well as the categorical variables of knowledge and health motivation (see Table 1a in the Appendix). Items in the HBM predictor categories were measured on a 5-point Likert-type scale, with the following possible responses: strongly agree (1), agree (2), neither agree nor disagree (3), disagree (4), and strongly disagree (5). Each scale was defined as a sum of separate questions, with the sign of a correlation coefficient between the question and the scale divided by the number of the questions.

### 4.3. Data Collection Procedure

The envelopes with the questionnaires and cover letters were randomly distributed among several departments, both in companies that had offered vaccination to their employees during 2006/2007 and in those that had not offered the vaccine. This procedure was carried out only after we asked the human resources department of each company to construct a sample made up of administrative as well as production employees, both genders and a range of ages.

A cover letter was attached to the self-administered questionnaire form explaining the purpose of the study. In addition, the cover letter explained that participation in the study was voluntary and provided details of the researchers as well as instructions to return the completed questionnaire in the enclosed envelope to the human resource department via interoffice mail (at each workplace where the human resources department gave formal permission to distribute the questionnaires to the employees on a voluntary basis). Two weeks later, we contacted the companies and collected the completed questionnaires. In addition, to increase the number of participants from the high-tech industries, we distributed questionnaires (with cover letters) to 81 high-tech workers that were studying at the MBA program in the Technion. A total of 879 questionnaires were distributed in the study, and 616 questionnaires were returned by the respondents, representing a response rate of 70.07%. This sample size provided power of 80% and more for the first main outcome (vaccination status in 2006–2007) for factors with OR 1.5 and more. For the second outcome (the intention to be vaccinated), this sample size provided the power of 80% for factors with OR more that 1.75 or 1.5 depending on the percent of exposed persons.

### 4.4. Data Analysis

The statistical package STATA 10 SE was used to conduct a statistical analysis of the data. Chi-square tests were used to determine how selected categorical (e.g., gender) variables, including demographic factors, were related to the two dependent variables: (a) vaccination status in 2006/2007, and (b) intention to be vaccinated in the coming year. For an easier and more instructive interpretation, we performed a binary logistic regression (and not ordinal). Therefore, we transformed the initial 5-point Likert scale of intention to be vaccinated into a binary one: the dependent variable is a dichotomous variable that is equal to one if an individual said that he/she “definitely intends” or “probably intends” to get a flu shot in the next year, and to zero for “definitely do not intend”, and “probably do not intend” (the answer “do not know” was excluded).

The statistical significance of the difference between the continuous variable means (e.g., age, summary scales, *etc.*) for two different groups (for example, for vaccinated *versus* non-vaccinated participants) was determined by t-test. Multiple logistic regressions were conducted to identify the impact of demographic factors, factors derived from the HBM model, and other factors of interest regarding intention to be vaccinated and vaccination status in 2006/2007.

## 5. Results

### 5.1. Descriptive Statistics

In 2006/2007, 24% of the respondents reported they had been vaccinated against influenza (145 out of 616). Almost 68% of the employees that reported getting vaccinated in 2006/2007 said they had been vaccinated at a worksite program. Others (32%) got the vaccine from their family physician or at their HMO clinic.

Table 1 summarizes the basic demographic information and characteristics for the sample according to vaccination status in 2006/2007. The table reveals that among the 568 participants (53% men and 47% women), percentage of vaccinated employees in 06/07 was 30% for men and 18% for women (p value < 0.01). In addition, the percentage of vaccinated employees was higher among married *versus* unmarried individuals (27% and 15%, respectively, p value < 0.01), and among veteran Israelis *versus* new immigrants (arrival after 1990) (p value = 0.02). The table also indicates an increase in vaccination rate with age (p value < 0.01) (49% among those aged 55 and over), an increase as the perceived cost of vaccination decreases (p value < 0.01), and an increase associated with increased perceived self-risk of contracting influenza without being vaccinated (p value < 0.01). Moreover, among the 252 employees offered flu vaccination at work, 39% were vaccinated in 2006/2007, while only 12% of the 310 who were not offered the vaccine at work were vaccinated (p value < 0.01). The percentage of those vaccinated did not differ significantly among those with higher and lower levels of education.

### 5.2. Main Reasons for Accepting or Rejecting Flu Shots

Table 2 summarizes the main reasons indicated for accepting (Table 2a) or rejecting (Table 2b) the flu shot in 06/07. The results in Table 2 part (a) show that the top motivators for getting a flu-shot in 2006/2007 were: (a) To reduce my chances of contracting influenza (80%); (b) The vaccine was available at my work place (35%); (c) Vaccination was recommended to me (19%); (d) I am accustomed to getting a flu shot each year (19%); (e) I do not want to miss any work because of influenza (19%). Respondents could select more than one reason.

Table 2 part (b) also shows that the main reasons for the decision not to take the vaccine in 2006/2007 were: (a) There are many strains of influenza (23%); (b) The vaccine is not effective (22%); (c) I do not believe in immunizations (21%); (d) I do not like injections (20%). In addition, it is interesting to note that 16% of the unvaccinated sample mentioned lack of time as one of the reasons for not getting vaccinated. Moreover, some of the reasons for rejecting the vaccine indicate a lack of knowledge about the vaccine among employees, including: “The vaccine is not effective” (22%); “The vaccine is not important” (18%); and “The vaccine can cause influenza” (8%).

### 5.3. Effect of Offering Vaccination at Workplace on Intention to Get Flu Shot

Table 3 shows intention to be vaccinated in the coming year if offered at work among employees who were not offered the vaccine at work the previous year. The results indicate that 36% percent of the 264 employees who were not vaccinated in 2006/2007 and not offered the vaccine at work said they intend to be vaccinated in the next year if the vaccine is offered to them at work. In addition, 30% of them said they are not sure whether or not they will get vaccinated next year if the vaccine is offered at work, though it is reasonable to assume that some of them will eventually get vaccinated.

Moreover, according to Table 3, 23% of the 133 employees who declared that in general they do not intend to be vaccinated during the next 12 months indicated that if the vaccine is offered at their place of work, they will be vaccinated during the coming year. In other words, these results indicate that the incentive to get vaccinated is substantially higher when the vaccine is available at workplaces than otherwise.

### 5.4. Results for HBM Categories

Table 4 shows the mean values of the HBM model categories and the category variables (defined in Appendix 1a) as indices on a 5-point Likert scale (the scale for HBM categories ranged from “strongly agree”-1, to “strongly disagree”-5) measured by vaccination status in 2006/2007 and by intention to be vaccinated. The Cronbach’s alpha coefficients for the HBM categories were: perceived susceptibility (HBM1) −0.654, perceived seriousness (HBM2) −0.628, perceived benefits (HBM3) −0.686, perceived barriers (HBM4) −0.723, and health motivation −0.601.

As expected, the results in Table 4 indicate that for individuals who had been vaccinated in 2006/2007, the levels of the following five categories were significantly lower than these levels for the non-vaccinated group: susceptibility (2.98 vaccinated, 3.2 non-vaccinated); seriousness (1.92 vaccinated, 2.15 non-vaccinated) benefits (2.56 vaccinated, 3.22 non-vaccinated), health motivation (2.56 vaccinated, 2.64 non-vaccinated), and knowledge (3.01 vaccinated, 3.43 non-vaccinated). The barriers category was significantly higher for the vaccinated than for the non-vaccinated group (3.89 and 3.26, respectively). Similar differences in HBM categories were obtained between the group that intends to be vaccinated the next year and the group that does not intend to get a flu shot in the coming year. Therefore, on average vaccinated individuals perceived influenza as a more serious illness than did those who were not vaccinated. In addition, vaccinated individuals felt they were more susceptible to illness, perceived more benefits from vaccination, and had fewer barriers to getting the flu shot than did the non-vaccinated employees. Moreover, on average the vaccinated individuals were more knowledgeable regarding the vaccine and influenza and had higher levels of health motivation. The same conclusions hold for the differences between the group of employees that intended to get the vaccine in the next year and the group that did not intend to do so. In general, these results are compatible with previous studies that referred to health care employees [13,15].

### 5.5. Results of the Analytical Model

The analytical model examines the effect of each one of the explanatory variables on the dependent variable, controlling for all other variables including the socio-demographic characteristics. Table 5 presents the results of the logistic model regressions. In Table 5(a), the dependent variable is a dichotomous variable that is equal to one if the individual had a flu shot in 2006/2007 and to zero if not. In Table 5(b), the dependent variable is a dichotomous variable that is equal to one if an individual said that he/she “definitely intends” or “probably intends” to get a flu shot in the next year, and to zero for “definitely do not intend”, and “probably do not intend”. The analysis of “intention to be vaccinated” was performed among those who did not take the flu shot in 2006/2007, since we found that the vaccination status in 2006/2007 was the strongest predictor of the intention to get the vaccine in 2008 (124 out of 141 subjects vaccinated in 2006/2007 said that they intend to get the flu shot in 2008, *versus* only 77 out of 455 that were not vaccinated in 06/07 and said that they intend to take it in 2008 (OR = 35.8 95%, CI = (19.9, 66.6)).

The independent variables in parts (a) and (b) are: age group (less than 41, 41–55, 56 and above), gender, health status, whether or not the vaccine was recommended to the individual, perceived cost of vaccination, whether or not the vaccine was offered at work, perceived infection risk without vaccination (high, medium, low), knowledge about influenza and the vaccine, and HBM categories.

The results in Table 5a (columns 3–5) show that the significant factors *positively* affecting vaccination status in 2006/2007 are: (a) whether the vaccine was offered at workplace: with vaccine offered at workplace increasing the odds of employees getting the vaccine by 5.7; (b) employee age: the odds of an employee aged 56 and up getting vaccinated are three-times higher than for an employee aged less than 41, (c) higher perceived risk of infection without vaccination, (d) HBM3—higher levels of perceived benefits, and (e) HBM4—lower levels of perceived barriers.

The results in Table 5b (columns 6–8) show that the same significant factors affect the intention to get the vaccine in the next year as those affecting vaccination status in 2006/2007 (except for the age group variable). In addition, we found that higher levels of perceived seriousness of influenza (HBM2) and greater knowledge about the illness and the vaccine increase the odds of intention to get the vaccine.

To test the robustness of the results, we analyzed equations (1) and (2) simultaneously, (meaning a joint analysis for the two dependent variables: the intention to be vaccinated and the status of vaccination in 2006–2007). The results of this analysis, not shown in the paper, indicated that the set of coefficients and significant predictors are very similar to the predictors that we found in the separate equations analysis.

Using additional logistic regression, we also examined the effect of past vaccination (number of vaccinations during the years 2002–2005) on vaccination status (data not shown here). The results indicate that the odds of being vaccinated increase significantly with higher perceived benefits, lower perceived barriers, vaccine offered at workplace, and higher number of vaccination during the years 2002–2005. Yet, we did not find any significant effect of perceived infection risk or of age group on employees’ vaccination status. These results may suggest that past experience with the vaccine dominates other possible reasons for deciding to get vaccinated, including age. People who had a good experience with the vaccine in the past may continue to be vaccinated routinely each year. Our result that past experience with the vaccine affects vaccination status is also compatible with the findings of Sendi *et al.* (2004).

## 6. Summary and Conclusions

Although the younger working population is less prone to serious illness following influenza infection, it has been shown that influenza vaccination may prevent illness, and therefore a transient loss in quality of life, and may reduce direct medical costs and productivity costs due to absence from work [8–10]. Workplace vaccination of healthy adults against influenza has had health benefits that translate into financial benefits for the employer, with cost savings significantly outweighing the costs of the vaccination program [4,6].

The current study examined the factors, and in particular workplace vaccination programs, affecting the decision to get vaccinated against influenza among employees in Israel. The results show that workplace vaccination programs significantly increase influenza vaccine compliance among employees. Offering the vaccine at worksites facilitates easier access to vaccination and reduces the overall costs, including the time wasted and the inconvenience of getting the vaccine at HMO clinics. In other words, a vaccination program reduces employee barriers to getting vaccinated.

Although vaccination programs significantly increase compliance rates (39% at workplaces with vaccination program *versus* 12% at workplaces without such a program), vaccination rates are still quite low in the sample of the current study. As our findings indicate, this relatively low rate stems from low perceived vaccination benefits, high barriers to getting a flu shot (e.g., worry about side effects), and lack of knowledge about influenza and the vaccine. In line with the findings of [24], our results also show that past experience with the vaccine is a significant factor in employees’ decisions to get a flu shot. It may be that people who have had good experience with the vaccine in the past will continue to be routinely vaccinated each year. Therefore, offering the vaccine at workplaces on a regular basis is important.

In the light of the current research results, the following recommendations can be made: (a) to encourage vaccination programs at workplaces and in other public places, such as shopping centers (during the relevant season) at days and times convenient for working people; (b) to consider offering the vaccine free of charge to the entire population, since recent behavioral economics research has shown that people strongly react to free products and services [25]; (c) to offer an advertising campaign stressing the importance of vaccination. Key strategies for the success of such a campaign include providing employees with evidence-based information related to influenza and immunization using a variety of media [26]. Goldstein *et al.*, 2004 suggested that tailored employee educational campaigns should target the primary reasons for noncompliance with vaccinations, “fear of side effects” and “perceived ineffectiveness of the flu vaccine”, which show insufficient knowledge about the vaccine’s effectiveness and few side effects [27]. These recommendations are also in line with the findings of Kimura *et al.*, (2007) [28], that the combination of a vaccine day at worksite and an educational campaign was most effective in increasing vaccine coverage of health care workers. In other words, multiple strategies used in concert will likely achieve higher vaccination rates than would single strategies alone. Finally, future study is important to focus on factors affecting compliance with essential vaccinations among different socio-demographic groups.

## Figures and Tables

**Table 1.** Comparison of sample characteristics by vaccination status in 2006/2007.

			**Vaccination status in 2006/2007**		
		**Number**	**No (%)**	**Yes (%)**	**p-Value**
Gender	Male	300	70	30	0.00
	Female	268	82	18	
Age group	18−40	279	84	16	0.00
	41−54	214	79	21	
	55 +	110	51	49	
Marital status	Married	451	73	27	0.00
	Unmarried	155	85	15	
Nationality	Jews	554	76	24	0.90
	Other	48	77	23	
Education	Secondary or below	122	82	18	0.13
	Tertiary	472	76	24	
New immigrants	Before 1990	136	71	29	0.02
	(after 1990)	44	89	11	
Five-year influenza vaccination status	never	337	100	0	0.00
	1−2 times	123	65	35	
	3 and above	105	12	88	
Vaccine offered at workplace	Yes	252	61	39	0.00
	No	310	88	12	
Perceived cost of vaccination	expensive	53	72	28	0.00
	cheap	164	65	35	
	Free of charge	32	56	44	
	Do not know	319	87	13	
Perceived risk of contracting influenza without vaccine	high	148	56	44	0.00
	Medium	334	81	19	
	Low	101	87	13	

**Table 2.** Main reasons for getting or rejecting flu shot in 2006/2007.

a. Reasons for getting flu shot* (N = 195)			b. Reasons for rejecting flu shot * (N = 483)		
Reasons for getting flu shot*	**Number of respondents selecting response**	**% of vaccinated employees selecting response**	Reasons for rejecting flu shot*	**Number of respondents selecting response**	**% of unvaccinated employees selecting response**
To reduce my chances of getting influenza	156	80	There are many strains of influenza	109	23
The vaccine was available at my work place	69	35	The vaccine is not effective	106	22
I do not want to miss any work because of influenza	38	19	Don’t believe in immunizations	100	21
I got a recommendation	37	19	Do not like injections	98	20
I am accustomed to getting a flu shot each year	37	19	The vaccine is not important	85	18
Not to transfer the illness to other people	35	18	No time to get the vaccine	79	16
The flu shot was free of charge	16	8	Potential side effect	74	15
I am over 65 and/or have a chronic illness	14	7	I am not afraid of influenza	71	15
I was afraid of Avian influenza	7	4	I do not need the vaccine since I do not suffer from chronic illness	58	12
Other reason	2	2	The vaccine can cause influenza	40	8

* Respondents could select more than one reason.

**Table 3.** Intention to get vaccinated if vaccine is offered at workplace, for those where vaccine was not offered at workplaces.

				Intend to get the vaccine if the vaccine is offered at workplace*		
		**N**	**%**	**Yes (%)**	**No (%)**	**Do not know (%)**
**Vaccination 06/07**	**No**	264	100	36	34	30
	**Yes**	36	100	75	14	11
**Intention to get vaccinated in the next 12 months**	**No**	133	100	23	18	59
	**Yes**	72	100	86	7	7

* For employees that were not offered the vaccine at work in 2006/2007.

**Table 4.** Mean values of Health Belief Model (HBM) measures by vaccination in 2006 or 2007, and by intention to be vaccinated in the next year.

Scale*	**Vaccinated**		**Non-vaccinated**		**t test (P value)**	**Intend to get vaccinated**		**Do not intend to get vaccinated**		**t test (P value)**
	**N**	**Mean (SD)**	**N**	**Mean (SD)**		**N**	**Mean (SD)**	**N**	**Mean (SD)**	
**Susceptibility**	141	2.98 (0.06)	461	3.20 (0.04)	2.95 (0.00)	200	2.90 (0.05)	244	3.33 (0.05)	5.30 (0.00)
**Seriousness**	143	1.92 (0.05)	467	2.15 (0.03)	3.60 (0.00)	200	1.90 (0.04)	245	2.24 (0.05)	5.08 (0.00)
**Benefits**	145	2.56 (0.06)	470	3.22 (0.03)	9.53 (0.00)	201	2.55 (0.05)	246	3.46 (0.04)	14.0 (0.00)
**Barriers**	142	3.89 (0.05)	461	3.26 (0.03)	−10.01 (0.00)	199	3.69 (0.05)	241	3.21 (0.04)	−6.56 (0.00)
**Health Motivation**	141	2.50 (0.06)	464	2.64 (0.03)	1.99 (0.04)	199	2.44 (0.04)	242	2.69 (0.04)	3.32 (0.00)
**Knowledge**	140	3.01 (0.07)	459	3.43 (0.04)	4.66 (0.00)	198	3.01 (0.06)	240	3.54 (0.05)	5.86 (0.00)

* The 5-point scale for the HBM categories ranged from “strongly agree” (1) to “strongly disagree” (5).

**Table 5.** Results of logistic regression for dependent variables: (a) vaccination status in 2006/2007, and (b) intention to get vaccinated in 2008 for those who did not take the vaccine in 2006/2007.

**Dependent variable**		**(a) Vaccination 06–07 (N = 538, Pseudo R^2^ = 0.40)**			**(b) Intention to get vaccinated (N = 395, Pseudo R^2^ = 0.43)**		
**Explanatory variables**							
		**OddsRatio**	**Std.Err.**	**P > |z|**	**OddsRatio**	**Std.Err.**	**P > |z|**
Age group Base (less than 41)	Age group 2 (41−55)	0.80	0.27	0.52	0.57	0.19	0.11
	Age group 3 (56+)	3.08	1.07	0.00	1.04	0.45	0.91
Gender (base = male)	Female	0.58	0.17	0.06	0.74	0.23	0.34
Perceived cost of vaccination		0.83	0.10	0.17	0.95	0.13	0.72
Vaccine recommendation (base = recommended)		1.21	0.34	0.50	1.21	0.35	0.50
Vaccination offered at work (base = not offered)	Offered	5.71	1.78	0.00	2.52	0.75	0.00
Perceived infection risk without vaccination (base = high risk)	Medium risk	0.38	0.12	0.00	0.39	0.14	0.01
	Low risk	0.31	0.15	0.01	0.09	0.05	0.00
Health status (base = good)	Not good	1.47	0.79	0.46	2.40	1.50	0.16
HBM1-Susceptibility**		0.88	0.18	0.56	1.04	0.25	0.86
HBM2-Seriousness**		0.80	0.16	0.27	0.65	0.13	0.04
HBM3-Benefits**		0.38	0.07	0.00	0.18	0.04	0.00
HBM4-Barriers**		3.52	0.89	0.00	2.43	0.64	0.00
Health motivation		0.85	0.16	0.41	0.68	0.14	0.07
Knowledge		0.82	0.12	0.20	0.69	0.11	0.03

** For covariates being considered as continuous, the OR is for increment of the variables by 1.

## Appendix

**Table 1a.** HBM categories and categorical variables*.

Variables		Statements
HBM Categories	Susceptibility	Working with many people each day increases my chances of getting the flu
		My chances of getting the flu are good
		I worry a lot about getting the flu
		I will get the flu next year
	Seriousness	Getting the flu would disrupt my family
		Having the flu would make daily activities more difficult
		Flu can be a serious disease
	Benefits	Getting a flu shot will prevent me from getting the flu
		Getting a flu shot will prevent me from missing work
		I would not be afraid of getting the flu if I got a flu shot
	Barriers	Getting a flu shot can be painful
		Getting a flu shot is time consuming
		There are too many risks in getting a flu shot
		I am concerned about having a bad reaction to the flu shot
Categorical variables	Health Motivation	I eat a well-balanced diet
		I follow medical orders because I believe they will benefit my state of health
		I search for new information related to my health
		I exercise regularly at least twice a week
	Knowledge	People often get sick from flu injections

* The 5-point scale for the categories ranged from “strongly agree” (1) to “strongly disagree” (5).
